# New York State Veterinary License Law

**Published:** 1895-12

**Authors:** 


### LEGISLATION.

### NEW YORK STATE VETERINARY LICENSE LAW.

As there seems to be some misunderstanding in regard to the requirements for registration to practise veterinary medicine in New York State since the new law went into effect July 1, 1895, we publish the following with the approval of the Regents and of the Board of Veterinary Examiners.

The registered veterinary colleges recognized by the Regents for and after July 1, 1895, are: The New York College of Veterinary Surgeons, New York; The American Veterinary College, New York; The Veterinary Department of the University of Pennsylvania, Philadelphia; The Veterinary School of Harvard University, Boston; The McKillip Veterinary College, Chicago; The Veterinary College, Ames, Iowa; The School of Comparative Medicine, McGill University, Montreal, Canada; the veterinary colleges of Great Britain under the Royal College of Veterinary Surgeons; the veterinary colleges of Continental Europe under government control.

Diplomas will be recognized in accordance with the following resolution, if issued before July 1, 1895:

*Resolved*, That diplomas granted by all legally incorporated veterinary schools be accepted for graduates prior to July 1, 1895, as meeting requirement five for admission to the licensing examination, viz.: A degree from a registered veterinary medical school, provided that the holders have studied veterinary medicine three full years, including three satisfactory courses in three different academic years, or can offer for the fourth requirement evidence of five or more years' reputable practice of veterinary medicine.

1. Veterinarians in practice in New York State who were imperfectly registered prior to July 1, 1895, but whose registration is not legal owing to error, misunderstanding, or unintentional omission, may apply, furnishing proper evidence, to the Regents, and on the unanimous recommendation of the Board of Veterinary Examiners they may receive from the Regents a certificate of the facts, which may be registered with any county clerk, and shall make valid the previous imperfect registration.

2. Those who have matriculated in a New York State veterinary medical school prior to July 1, 1896, and who receive a veterinary degree from a registered veterinary medical school before July 1, 1897, may, without further examination, on payment of ten dollars to the Regents, and on submitting such evidences as they may require, receive from them an indorsement of their licenses or diplomas conferring all rights and privileges of a Regents' license issued after examination.

3. Students matriculating in a veterinary medical school before October 1, 1895, and receiving their diplomas before July 1, 1898, are exempt from the preliminary Regent's examination.

